# The Versatile Roles of the tRNA Epitranscriptome during Cellular Responses to Toxic Exposures and Environmental Stress

**DOI:** 10.3390/toxics7010017

**Published:** 2019-03-25

**Authors:** Sabrina M. Huber, Andrea Leonardi, Peter C. Dedon, Thomas J. Begley

**Affiliations:** 1 Department of Biological Engineering, Massachusetts Institute of Technology, Cambridge, MA 02139, USA; smhuber@mit.edu or sabrina.huber@hest.ethz.ch (S.M.H.); pcdedon@mit.edu (P.C.D.); 2 RNA Institute, State University of New York, Albany, NY 12222, USA; aleonardi2@albany.edu; 3 College of Nanoscale Science and Engineering, State University of New York, Albany, NY 12203, USA; 4 Antimicrobial Resistance Interdisciplinary Research Group, Singapore-MIT Alliance for Research and Technology, Singapore 138602, Singapore; 5 Department of Biological Sciences, State University of New York, Albany, NY 12222, USA

**Keywords:** epitranscriptomics, tRNA modifications, stress response mechanisms, codon-biased translation

## Abstract

Living organisms respond to environmental changes and xenobiotic exposures by regulating gene expression. While heat shock, unfolded protein, and DNA damage stress responses are well-studied at the levels of the transcriptome and proteome, tRNA-mediated mechanisms are only recently emerging as important modulators of cellular stress responses. Regulation of the stress response by tRNA shows a high functional diversity, ranging from the control of tRNA maturation and translation initiation, to translational enhancement through modification-mediated codon-biased translation of mRNAs encoding stress response proteins, and translational repression by stress-induced tRNA fragments. tRNAs need to be heavily modified post-transcriptionally for full activity, and it is becoming increasingly clear that many aspects of tRNA metabolism and function are regulated through the dynamic introduction and removal of modifications. This review will discuss the many ways that nucleoside modifications confer high functional diversity to tRNAs, with a focus on tRNA modification-mediated regulation of the eukaryotic response to environmental stress and toxicant exposures. Additionally, the potential applications of tRNA modification biology in the development of early biomarkers of pathology will be highlighted.

## 1. Introduction

In an ever-changing environment, living systems are subjected to stresses such as temperature fluctuations, nutrient limitations, and exposures that damage intracellular biomolecules. Cells respond to these stresses by activation of response, repair, and adaptation pathways or, if the damage is too severe, by initiation of cell death systems. Among the well-studied stress response mechanisms are the heat shock, unfolded protein, DNA damage, oxidative, and nutrient stress responses, which are reviewed in detail elsewhere [1,2,3]. The molecular underpinnings of these mechanisms have been extensively explored at the level of signaling pathways, changes in transcription, and the proteome. Here, we focus on recently emerging mechanisms of cellular stress response involving the dynamic regulation of ribonucleoside modifications that control transfer RNA (tRNA) metabolism, structure, and function, which, in turn, modulate protein translation and a cell’s ability to cope with stress. We discuss how tRNA modifications can affect tRNA stability, maturation, and codon recognition in mRNA, all of which regulate cell survival and adaptation during environmental stress and xenobiotic exposures. Aberrant modification of tRNAs is also directly linked to human diseases, including neurological disorders, metabolic diseases, and cancer [4,5,6]. As such, tRNA-based stress response mechanisms have the potential for diagnostic applications and could be useful biomarkers, as they have been shown to be linked to increased levels of reactive oxygen species (ROS), mitochondrial dysfunction, DNA damage, and changes in metabolism [7,8,9,10,11,12]. 

## 2. tRNA-Based Regulation of Cellular Processes

tRNAs are best known as the adaptor molecules of the translation machinery, in which they deliver the appropriate amino acids to the ribosomes according to the interaction of their anticodons with the codons of messenger RNAs (mRNAs). tRNAs are extensively post-transcriptionally modified by epitranscriptomic writer enzymes, with these RNA modifications having structural and functional roles, as well as playing downstream roles in different biological processes. A detailed description of these RNA modifications is given in Section 3. Beyond their role as adaptors in protein synthesis, tRNAs have been shown to be critically involved in many other cellular processes. Aminoacylated-tRNAs (aa-tRNAs) act as amino acid donors not only to nascent peptide chains during translation, but also to membrane lipids, to peptidoglycan precursors, and to the amino-terminus of proteins [13]. In addition, tRNAs act as biosynthetic precursors of antimicrobial molecules and tetrapyrroles [13,14]. Uncharged tRNAs are used by retroviruses to prime DNA synthesis and are thus essential for viral replication [15,16,17]. During the priming of DNA synthesis in human immunodeficiency virus type 1 (HIV-1) infections, the 3′-end of the primer tRNA is complementary to a region of the viral RNA called the primer binding site [18]. 

tRNAs also function as stress sensors and are key for initiating stress responses [19]. For example, eukaryotic cells respond to nutrient deprivation by global inhibition of protein synthesis, which can be mediated by tRNAs acting as signaling molecules. The limited availability of extracellular amino acids during starvation leads to the accumulation of uncharged cytosolic tRNAs that directly bind to the protein kinase GCN2 [20,21]. This tRNA binding activates the GCN2 kinase activity and results in the phosphorylation of elF2, which in turn regulates translation in amino acid-starved cells and promotes increased synthesis of the transcription factor GCN4 that activates amino acid biosynthetic genes. Interestingly, the GCN4-regulated transcripts exhibit a codon bias that can be linked to regulation by modified wobble uridines in tRNA [22], as discussed shortly. Furthermore, cytosolic and mitochondrial tRNAs can inhibit apoptosis by binding to cytochrome c, thereby blocking the formation of the apoptosome [23,24]. Furthermore, tRNAs serve as a source of small noncoding RNAs called tRNA-derived fragments (tRF), which vary in length, biogenesis, sequence, and function [25,26]. Examples of functions of tRFs in the literature include the repression of gene expression in a miRNA-like fashion, inhibition of translation through ribosome binding, and modulation of cell proliferation [25,27,28,29]. Interestingly, the formation of tRFs can be controlled by ribonucleoside modifications which is discussed in more detail in Section 4.3.

Here we explore how tRNA modifications play a role in this tRNA-based regulation of cellular processes in the face of stressful conditions.

## 3. Ribonucleoside Modifications

**Figure 1:**
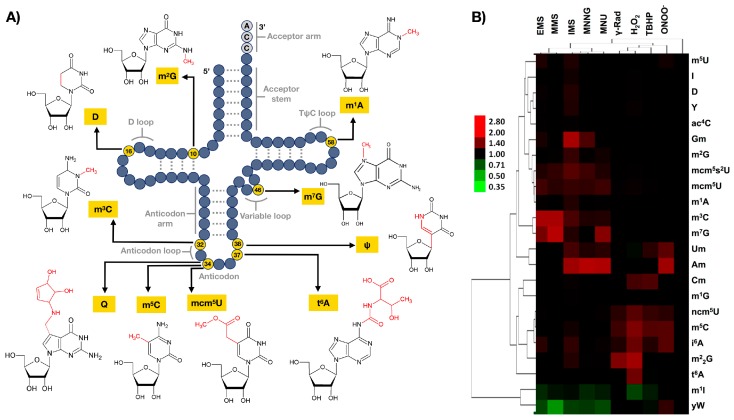
(**A**) Clover-leaf structure of eukaryotic tRNA formed through base pairing in the acceptor stem, D-loop, anticodon arm, and TψC-loop. Structures and positions of some modifications discussed in the review are indicated. (**B**) Hierarchical cluster analysis of average fold-change values for tRNA modifications in total tRNA from *S. cerevisiae* exposed to equitoxic (LD_80_) doses of various alkylating (EMS, ethyl methansulfonate; MMS, methyl methansulfonate; IMS, isopropyl methanesulfonate; MNNG, *N*-methyl-*N*′-nitro-*N*-nitrosoguanidine; MNU, *N*-nitro-*N*-methylurea) and oxidizing agents (γ-Rad, γ-radiation; H_2_O_2_, hydrogen peroxide; TBHP, tert-butyl hydroperoxide; ONOO^−^, peroxynitrite). Yeast cells were exposed to LD_80_ doses of the agents and tRNA modifications were quantified by liquid chromatography—tandem mass spectroscopy (LC-MS/MS). The fold-change values were derived from the average of normalized MS signal intensity data from five biological replicates relative to unexposed controls, and hierarchical clustering analysis was performed in log space (log_2_) and visualized as a heat map. Reproduced from [11], ACS publications, 2015.

Post-transcriptional RNA modifications were first described in 1957, when the presence of pseudouridine was demonstrated in yeast RNA extracts [30]. Since then, more than 140 chemically distinct ribonucleosides have been characterized, some of which are conserved throughout all domains of life. Modified ribonucleosides occur in all RNA classes, with tRNAs containing the most numerous and chemically diverse modifications [31,32]. On average, ~17% of the 80–90 ribonucleosides in tRNA are modified, ranging from relatively small structural changes, such as methylation or acetylation, to highly intricate chemical alterations that give rise to so-called hypermodified bases such as queuosine (Q) (Figure 1A). The type, location, and abundance of modifications not only vary between different tRNAs, but also differ within similar isoacceptors (tRNAs bearing the same amino acid but different anticodons). Ribonucleoside modifications are located throughout tRNA molecules and can affect tRNA stability, folding, localization, transport, processing, and function [33]. The anticodon loops of nearly all tRNAs are heavily modified, predominantly at positions 34 and 37 [31,32]. The ribonucleotide at the wobble position 34 pairs with the third mRNA codon base in the aminoacyl-tRNA binding site (A-site) of the ribosome during decoding and thus is crucial for accurate reading of the genetic code. Modifications at the wobble position of the tRNA anticodon have been shown to allow decoding of multiple mRNA codons differing by the third nucleoside (synonymous codons) or restrict pairing with noncognate codons [34]. Position 37 in tRNA is adjacent to the 3′-side of the anticodon and has been shown to prevent frame shifting during translocation by stabilizing codon–anticodon interactions [35]. It is important to note that tRNA modifications play an important role during HIV-1 infections. There is an additional interaction between an A-rich loop located upstream of the primer binding site region and the lysine tRNA anticodon loop dependent on a sulfur in the wobble base modification 5-methoxycarbonylmethyl-2-thiouridine (mcm^5^s^2^U). This demonstrates the importance of modified ribonucleosides in tRNA function [18]. There is also evidence that tRNA modifications play important roles in organelle function. For example, decreased tRNA modification has been directly linked to the mitochondrial diseases myoclonus epilepsy associated with ragged-red fibers (MERRF) and mitochondrial encephalopathy, lactic acidosis, and stroke-like episodes (MELAS) [8,36,37]. These diseases are caused by a mutation in mitochondrial tRNA genes, with the change in sequence preventing formation of the tRNA modifications s^2^ and 5-taurinomethyluridine (tm^5^U), both disturbing codon–anticodon interactions to disrupt protein synthesis [37,38]. As mitochondrial translation is used to synthesize key enzymes involved in metabolism, the resulting mistranslation defects can alter proteins involved in the electron transport chain and ATP synthesis. The resulting defect in energy production can be linked to the muscle weakness and neurological dysfunction associated with MERRF and MELAS, and it provides a mechanistic link between tRNA modifications and energy metabolism.

### 3.1. tRNA Modification as Dynamic Marks

RNA modifications were long considered to be static and stable marks after their post-transcriptional enzymatic introduction by “writer” proteins and their removal from the transcriptome was thought to occur passively via degradation of the modified RNA followed by transcription of new unmodified RNA. However, the findings that exposure of wild type yeast to various alkylating and oxidizing agents caused signature patterns of changes in the relative quantities of numerous modified ribonucleosides in tRNAs (Figure 1B) [9,11], demonstrated the responsiveness of modified ribonucleosides to environmental cues and highlighted the potential for reversibility. The pattern of up- and downregulation of these modifications were unique to each stressor and predictive of exposure. For example, in response to the alkylating agents, mcm^5^U, mcm^5^s^2^U, m^3^C, and m^7^G were all increased, but were relatively unchanged in response to oxidizing agents. In contrast m^5^C, i^6^A, ncm^5^U and, to some extent, m^2^_2_G, were increased in response to oxidizing agents, but show little change in response to alkylating agents.

**Figure 2:**
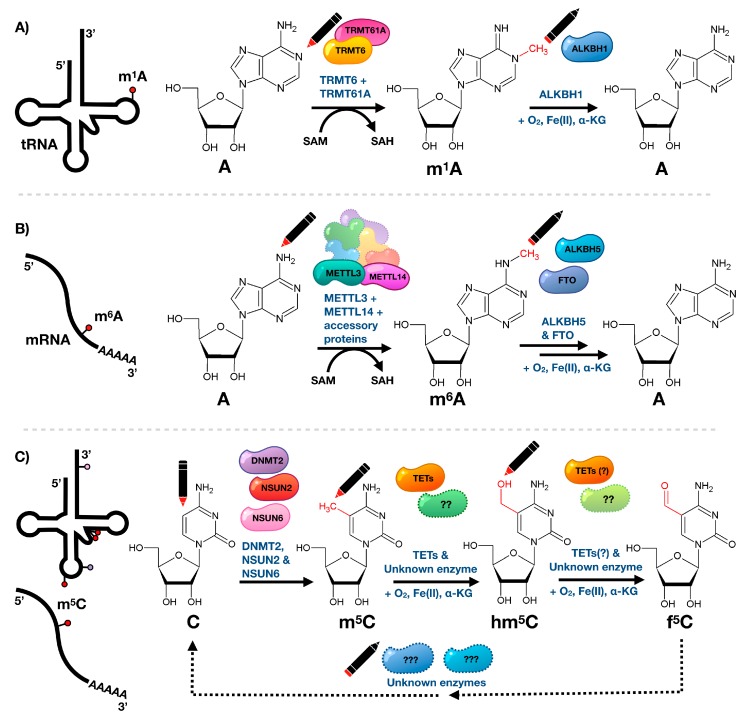
Reversibility and dynamics of RNA modifications. (**A**) The epitranscriptomic writer for the tRNA m^1^A modification is a TRMT6/TRMT61A complex, which uses S-adenosyl methionine (SAM) as a methyl donor. ALKBH1 removes the methyl group and requires oxygen, iron and α-ketoglutarate cofactors for demethylase activity. (**B**) N^6^-Methyladenosine (m^6^A) is added to mRNA via the METTL3-METTL14 heterodimer, which along with accessory proteins forms the N^6^-methyltransferase complex using SAM as methyl donor. The modification is removed by ALKBH5 or FTO requiring oxygen, iron, and α-ketoglutarate cofactors. (**C**) 5-Methylcytidine (m^5^C) on cytosolic tRNAs is added by DNMT2, NSUN2, and NSUN6, while m^5^C on mRNA is introduced by NSUN2 only. It undergoes further oxidative metabolism mediated by TET enzymes (and possible other unknown enzymes) to 5-hydroxymethylcytidine (hm^5^C), requiring oxygen, iron, and α-ketoglutarate. Further TET activity results in formation of 5-formylcytidine (f^5^C) using hm^5^C as precursor. These modifications have not been shown to be fully reversible although it is predicted that there are erasers for m^5^C, or its metabolites [46,47,48].

Technological advances throughout recent years lead to the identification of a number of “eraser” enzymes that are able to catalyze the active removal of modified residues, in particular methylation marks, and opened the door for dynamic regulation of modified ribonucleosides in tRNA (Figure 2). The reversibility of tRNA modifications, via the eraser ALKBH1, was recently shown to mediate the demethylation of m^1^A in tRNAs [39]. Notably though, reversibility was first shown in 2011, when the fat mass and obesity-associated protein (FTO) and ALKBH5 were both shown to robustly remove N^6^-methyladenosine (m^6^A) on polyadenylated RNAs in vitro through an oxidative demethylation mechanism and contribute to m^6^A levels in cellular mRNA (Figure 2) [40,41,42]. Since then, reversible N^6^-adenosine methylation has been demonstrated to play key roles in a number of biological processes, including mRNA nuclear export, the association of nuclear speckle proteins, splicing, cap-independent translation, UV-induced DNA damage response, leukemogenesis, and drug response [42,43,44,45]. 

Interestingly, recent studies show that in vivo FTO preferentially targets N^6^-2′-*O*-dimethyladenosine (m^6^A_m_), a highly prevalent mRNA modification found adjacent to the N^7^-methylguanosine cap at the first encoded nucleotide position, with nearly 100-times greater catalytic activity compared to m^6^A [49,50]. Using a transcriptome-wide map of m^6^Am, it was shown that the presence of this modification in the extended cap confers increased mRNA stability by reducing the susceptibility to DCP2-mediated decapping, thus influencing mRNA abundance and protein synthesis [49]. The application of stable isotope metabolic tracing demonstrated that oxidative processing is not limited to methylated adenosine residues and that 5-methylcytosine in RNA undergoes similar oxidative metabolism via 5-hydroxy- and 5-formylcytosine [47]. While the ten-eleven translocation (Tet) family of Fe(II)- and α-ketoglutarate-dependent dioxygenases, well known for their oxidation of m^5^C in DNA, have been shown to also promote the formation of hm^5^C in RNA [46], there is substantial evidence that TETs are not the only/main family of enzymes able to catalyze the oxidation of m^5^C in RNA. For instance, significant amounts of hm^5^C are not only found in TET triple knockout mouse embryonic stem cells but also in organisms that do not express any TET enzymes, such as *C. elegans* [46,47]. In support of this hypothesis, the formation of 2′-*O*-methyl-5-hydroxymethylcytidine (hm^5^Cm), a modification closely related to hm^5^C, has been shown to be TET-independent [48]. It remains unclear whether hm^5^C is a metabolic intermediate in the demethylation pathway and dynamic regulation of m^5^C, or whether it is an epitranscriptomic mark with its own function. The involvement of hm^5^C in an active demethylation pathway is supported by metabolic labeling studies in human embryonic kidney 293 cells that demonstrated that in small RNAs (< 200nt) hm^5^C itself or hm^5^C-containing transcripts are subject to enhanced turnover [48]. However, sucrose gradient fractionation followed by dot blotting in *Drosophila* S2 cells revealed that mRNAs heavily loaded with ribosomes have a high hm^5^C content, suggesting that the function of RNA hydroxymethylation is to promote mRNA translation in vivo [51]. While RNA methylations have been at the forefront of dynamic examples of ribonucleoside modifications, it is likely that other examples of dynamically-regulated or reversible modifications will follow. These initial examples of RNA demethylases point towards the responsiveness of RNA modifications to environmental stimuli that allow organisms to react and adapt to changing environments. 

### 3.2. tRNA Modifications Prevent Translational Infidelity and Proteotoxic stress

Proper anticodon–codon pairing and maintaining the correct reading frame on translated mRNA are key functions linked to tRNA modifications. Studies specific to wobble uridine U34-based modifications and their corresponding writers have been published highlighting how the C5 and C2 position on U34 play key roles in preventing protein synthesis errors. For example, the writer tRNA methyltransferase 9 (Trm9) from yeast completes the formation 5-methoxycarbonylmethyluridine (mcm^5^U) and mcm^5^s^2^U by adding the terminal methyl group. The mcm^5^U and mcm^5^s^2^U modifications are found at U34 on tRNAs that decode arginine, glutamine, glutamic acid, and lysine. The arginine codons AGA and AGG are found in a split codon box with the codons AGU and AGC for serine. The mcm^5^U modification is needed to prevent pairing of tRNA^Arg^, which normally decodes AGA and AGC, with the AGU and AGC codons for serine. As such, cells deficient in Trm9 and mcm^5^-based modifications show increased levels of arginine misincoporation at serine codons [52]. In addition, *trm9Δ* cells show increased −1 frameshifts and activation of heat shock and unfolded protein response (UPR) pathways. Studies in yeast using cells deficient in the wobble uridine writers for s^2^ (Ncs2) and c^5^ (elp6) have employed a novel reporter system and ribosome profiling to demonstrate that there is increased proteotoxic stress due to tRNA modification defects and perturbed translation [53]. Additional studies in yeast have also demonstrated that wobble U tRNA modifications play important roles in maintaining reading frame, with unmodified or under modified tRNA not entering the ribosome A-site efficiently [54,55]. As the levels of mcm^5^U and mcm^5^s^2^U change during the cell cycle and in response to specific exposures [52], there could be dynamic changes in translational fidelity during stress responses. 

### 3.3. tRNA Modification Enzymes as Essential Features of the Cell Stress Response

Many genes encoding tRNA-modifying enzymes are essential for cell function, with losses causing severe defects in growth and development [56,57]. However, despite their conservation throughout evolution, many genes encoding tRNA modification enzymes are not essential under normal growth conditions. tRNA normally contains many modifications and is considered to be a stable RNA. Notably though, the loss of key epitranscriptomic marks can destabilize tRNA and lead to rapid degradation. For example, mature tRNA for valine missing m^5^C and m^7^G, specific to tRNA methyltransferase (Trm) 4 and Trm8, is surveyed and targeted by the rapid tRNA decay (RTD) pathway [58,59]. The ability to generally function in the absence of a tRNA modification is illustrated by the fact that tRNAs lacking modifications (e.g., in vitro transcribed tRNAs) still function in translation and that cells can compensate for lost modifications by increasing tRNA copy numbers to drive translation [60,61]. The absence of a modification often causes subtle phenotypic effects in cells, so it has been challenging to elucidate the exact biological functions of more than a few modifications. However, the deletion of writer proteins often increases the cellular sensitivity to specific stresses, demonstrating the importance of RNA modifications in adaptation to environmental changes. For instance, depletion of N^1^-methyladenosine (m^1^A) in the hyperthermophilic bacterium *Thermus thermophilus* resulted in a thermosensitive phenotype, suggesting a role of m^1^A in temperature adaptation [62]. In the same organism, loss of pseudouridine at tRNA position 55 (ψ55) caused abnormal increases in the levels of other modified nucleosides (Gm, m^5^s^2^U, and m^1^A) and led to growth retardation at lower temperatures [63]. An emerging literature now documents the critical roles of tRNA modifications in the cellular response to physiological changes, environmental changes, and stressful exposures [12,64,65,66].

## 4. Mechanisms by which tRNA Modifications Function in the Cell Stress Response 

### 4.1. m^1^A Affects Translation Initiation during Cell Stress 

**Figure 3:**
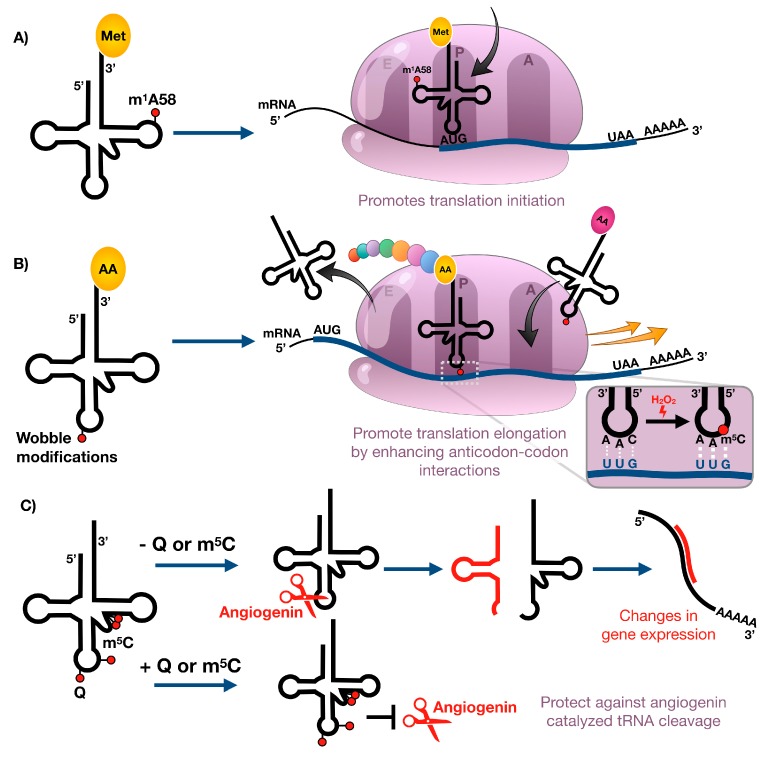
Stress-induced changes in tRNA modification levels can regulate (**A**) translation initiation, (**B**) translation elongation, and (**C**) tRNA cleavage.

Initiation of protein synthesis in eukaryotes is mediated by eukaryotic initiation factors (eIFs) and involves the assembly of the initiator tRNA, the 40S, and 60S ribosomal subunits into an 80S ribosome at the start codon of mRNAs, typically AUG coding for methionine [67]. The initiator methionyl-tRNA (tRNA^iMet^) is used exclusively during initiation of protein synthesis and is different from the elongator methionyl-tRNA, which is solely used for insertion of methionine into a growing polypeptide chain [68]. Initiator tRNAs carry a highly conserved N^1^-methyladenosine residue at position 58 (m^1^A58) that is key for the formation of a tertiary substructure not seen in elongator tRNAs (Figure 1A and Figure 3A) [69]. In yeast, m^1^A58 is essential for viability. Impaired function of m^1^A methyltransferases resulted in growth arrest and ultimately cell death, which could be attributed to rapid and specific degradation of mature tRNA^iMet^ in the absence of m^1^A58 by the use of pulse-chase experiments [70,71]. Hence, m^1^A58 provides direct means of regulating the intracellular levels of tRNA^iMet^ and thus the initiation of protein synthesis without affecting the elongation step of translation. The eraser ALKBH1 was recently shown to mediate the demethylation of m^1^A in tRNAs, providing evidence of reversible tRNA methylation [39]. Interestingly, glucose deprivation of HeLa cells resulted in increased expression of ALKBH1 which correlated with decreased levels of m^1^A and attenuated protein synthesis [39]. The levels of the m^1^A58 methyltransferase heterodimer Trmt6/Trmt61 showed no significant changes [39], suggesting the presence of an active demethylation pathway of m^1^A under glucose starvation. These studies provide the first evidence that reversible N^1^-adenosine methylation is involved in the control of protein synthesis in response to nutrient availability. 

### 4.2. Wobble tRNA Modifications Regulate Codon-Biased Translation of Stress Response Proteins

Post-transcriptional modifications at the wobble position in the anticodon loop of transfer RNAs have direct means to influence the decoding of the genetic code by mediating codon–anticodon interactions. Depending on the type of chemical modification, the interaction with certain codons is preferred due to increased stabilization of specific base pairs. tRNA wobble modifications should be able to regulate the decoding rates of synonymous codons which differ by the third nucleoside, with dynamic changes in modification levels regulating translation. Using a unique bioanalytical platform, we have shown that stressors cause the reprogramming of dozens of modified ribonucleosides in tRNA that regulate the selective translation of codon-biased mRNAs of critical stress response proteins required for cell survival [11,72]. For example, exposure of yeast to the oxidizing agent hydrogen peroxide (H_2_O_2_) caused an increase in 5-methylcytosine (m^5^C) found in the wobble position of leucine tRNA that reads UUG (1 of six leucine codons) (Figure 1). Codon reporter systems and proteomic studies have linked m^5^C to the enhanced translation of UUG-enriched mRNAs for oxidative stress response genes (Figure 3B). The role of m^5^C-based translation of UUG codons in cell survival was demonstrated when loss of the writer tRNA methyltransferase 4 (Trm4), catalyzing the formation of wobble m^5^C, rendered the cells sensitive to H2O2 exposure. Similar results have been shown for writers of mcm^5^U and mcm^5^Um modifications in yeast and mouse models, as deficiencies lead to sensitivity to alkylating and oxidizing agents, respectfully [12,73,74]. In both cases, codon specific reporter constructs, transcript- and protein-based studies, and increased levels of wobble uridine modifications in response to stress have supported the idea that stress promotes the translation of codon specific transcripts, which is coordinated by epitranscriptomic reprogramming. A similar phenomenon has been shown to occur in mycobacteria exposed to hypoxia [75]. This stress led to increased steady-state levels of proteins derived from ACG-enriched genes, with the increase dependent upon stress-induced conversion of mo^5^U to cmo^5^U at the wobble position of the UGU anticodon on the threonine tRNA that reads the ACG codon (Figure 3B).

### 4.3. tRNA Modifications Restrict Stress-Induced tRNA Cleavage

A third role for modified nucleosides in tRNA involves regulation of tRNA degradation and cleavage of tRNAs into small regulatory RNA fragments (Figure 3C). The latter is illustrated by angiogenin-mediated endonucleolytic cleavage of tRNAs in the anticodon loop, which is a widely conserved oxidative stress response in eukaryotes [76,77,78]. One example of modification-dependent angiogenin cleavage of tRNA under stress involves 5-methylcytidine (m^5^C). In nuclear-encoded eukaryotic tRNAs, m^5^C commonly occurs at six cytidine positions, namely, C34 and C38 in the anticodon loop; C48, C49, and C50 in the variable region; and C72 in the acceptor stem [79]. Cytosine-C5 methylation in mitochondrial encoded tRNAs is restricted to the variable loop and the acceptor stem and appears at C48, C49, and C72 [80]. Introduction of methyl groups to these sites is mediated by several members of a large protein family of conserved RNA m^5^C-methyltransferases, namely tRNA aspartic acid MTase 1 (TRDMT1, also known as DNMT2) and the NOP2/Sun domain proteins (NSUN) 2 and 6 [79,81]. While TRDMT1 specifically methylates position 38 in glycine, aspartic acid, and valine tRNAs [76,82], NSUN6 is responsible for the methylation of position 72 in threonine and cysteine tRNAs [81]. NSUN2 has wider substrate specificity and methylates the vast majority of tRNAs at positions 34, 48, 49, and 50 [83,84]. Studies by Blanco et al. revealed that the absence of NSUN2-dependent m^5^C sites in the variable loop leads to increased tRNA cleavage by the endonuclease angiogenin and the accumulation of 5′ tRNA-derived small RNA fragments in mouse models and dermal fibroblasts obtained from patients with Dubowitz-like syndrome [85]. These 5′ tRNA fragments induce cellular stress responses that lead to reduced protein translation rates, decreased cell size, and increased cell death in vitro and in vivo causing a syndromic disorder characterized by growth and neurodevelopmental deficiencies [85]. Similar to NSUN2-mediated methylation in the variable region, DNMT2-mediated cytosine-C5 methylation in the anticodon loop can also protect tRNAs from endonucleolytic cleavage by angiogenin under stress in *Drosophila* [76]. The prominent role of Dnmt2 in the stress response is further confirmed by the fact that *Drosophila* Dnmt2 mutants show significantly reduced viability under oxidative or heat stress [76]. While the presence of m^5^C limits the fragmentation of tRNA at various locations under stress, it is currently unclear how cytidine-C5 methylation modulates the activity of stress-induced endonucleases. The lack of m^5^C could result in a more flexible tRNA structure in which the anticodon loop becomes more exposed to tRNA cleavage enzymes. Alternatively, the modification could mask the sequence recognition motif of these enzymes.

m^5^C undergoes oxidative processing to hm^5^C [46,47,48]. It has been shown by quantitative isotope dilution-mass spectrometry that hm^5^C is enriched in tRNA fractions of HEK293T cells [48]. This provides evidence that m^5^C is dynamically controlled in tRNAs. While it is currently unclear whether hm^5^C also protects tRNAs against stress-induced cleavage, metabolic labeling studies have shown that hm^5^C is subject to enhanced turnover in RNA either due to specific tRNA degradation/cleavage or reversibility to unmodified C [48]. The oxidation of m^5^C to hm^5^C could provide means of dynamically regulating the cleavage potential of tRNAs under changing environmental conditions. Further studies should address the exact positions of hm^5^C and how this oxidative derivative of m^5^C affects angiogenin-mediated cleavage of tRNAs. 

Another example of a link between tRNA modifications and stress-induced tRNA cleavage involves queuosine restriction of tRNA cleavage during oxidative stress. Queuosine (Q) is a hypermodified residue found at the wobble position of tRNAs with GUN anticodons, namely histidine, asparagine, tyrosine, and aspartic acid (Figure 1A) [86]. While bacteria can synthesize Q de novo by a complex biosynthetic pathway [87], eukaryotes lack its synthesis pathways and rely on the uptake of the micronutrient queuine from dietary sources and the gut microbes, for subsequent enzymatic incorporation into tRNA [88,89]. Since Q-deficient mice do not exhibit any pathological symptoms in a stress-less environment, it has been suggested that the role of Q may be to protect the organisms against stress. A link between Q and the oxidative stress response in mice was established when it was shown that exogenous administration of queuine to mice with Dalton’s lymphoma ascites transplanted (DLAT) tumors improved the activities of antioxidant enzymes, such as catalase, superoxide dismutase, and glutathione peroxidase [90]. While this promotion of the antioxidant defense system could be evoked by queuine itself or by Q-modified tRNA, Wang et al. recently showed that Q-deficient HEK293T and HeLa cells produce significantly more tRNA halves from tRNA^His^ and tRNA^Asn^ upon arsenite stress and angiogenin treatment (Figure 3), suggesting that Q directly protects its cognate tRNAs against ribonuclease cleavage [91]. The total tRNA pool is not altered. In mammals, Q can be further modified by an unknown glycosyltransferase by addition of a mannosyl or galactosyl group to yield manQ or galQ, respectively [92]. The current lack of high-throughput methods for the simultaneous detection and quantification of Q, manQ ,and galQ from limited starting material, has hampered the studies of the Q-derivatives and thus their exact physiological roles remain poorly understood. 

Modifications do not always restrict the cleavage of tRNAs. For instance, wobble mcm^5^s^2^U is a target for the eukaryotic γ-toxin secreted by Kluyveromyces lactis killer strains and promotes the cleavage of tRNAs, causing irreversible growth arrest of sensitive yeast cells [93]. The importance of modified ribonucleosides is not limited to the eukaryotic host defense response. Colicin E5 and PrrC are *E. coli* endoribonucleases that specifically cleave Q- and 5-methylaminomethyl-2-thiouridine (mnm^5^s^2^U)-modified tRNAs, respectively [94,95]. These examples demonstrate that tRNA modifications can be critical determinants in defending host cells from the invasion of viruses or biotic stresses.

### 4.4. tRNA Modifications Affect tRNA Maturation during Stress 

Eukaryotic tRNAs are initially transcribed as larger precursors (pre-tRNAs) that require a variety of post-transcriptional alterations to become fully mature and functional tRNAs. These processing steps include the removal of the 5′-leader and 3′-trailer sequences, addition of the nucleotides CCA to the 3′-end, intron splicing, and introduction of a large number of ribonucleoside modifications [96,97]. Using Northern blot analysis and RNA sequencing, it was shown that tRNA maturation is differentially regulated during temperature and nonfermentable carbon source stress in yeast. Accumulation of aberrant tRNA precursors was observed upon shifting yeast to elevated temperatures and/or to glycerol-containing medium [98]. Interestingly, several tRNA modifications are added at the pre-tRNA stage [97,99], ensuring proper folding. For instance, studies in *Xenopus oocytes* showed sequential addition of base modifications during tRNA tyrosine maturation [100]. While in this particular organism, m^1^A, ψ, and m^5^C already occur in the pre-tRNA with immature 5′-leader and 3′-trailer sequences, m^2^_2_G, m^2^G, and D are introduced after maturation of the 5′- and 3′-termini but before intron splicing [100]. The order and location of incorporation of some modifications can be species-specific. For instance, wobble inosine modifications are incorporated into pre-tRNAs in the nucleus in human tRNAs and into mature tRNAs in the cytosol in *Trypanosoma*, respectively [101,102]. These findings demonstrate that tRNA modifications are stringently coupled with tRNA processing and maturation and suggest that environmental cues can affect tRNA precursor forms by controlling tRNA modification levels in a tRNA- and condition-specific manner.

## 5. RNA Modifications as Potential Biomarkers of Exposure and Disease Pathology

Based on these diverse roles for tRNA modifications in the cell stress response, it is reasonable to propose that ribonucleoside modifications can serve as biomarkers of specific stresses and environmental changes. Support for this idea comes from the observed role of tRNA modifications as sensors for changes in environmental and intracellular conditions. For instance, mitochondrial t^6^A is sensitive to intracellular CO_2_ [103]. The growth of HEK293T cells in sodium bicarbonate-free medium in the absence of CO_2_, caused a significant decrease in the frequency of t^6^A in mitochondrial tRNAs (mt-tRNAs), which could be rescued by the addition of sodium bicarbonate to the cell culture medium [103]. As a result, Lin et al. speculated that hypoxic conditions in solid tumors could affect t^6^A formation as mitochondrial CO_2_ is predominantly provided by the TCA cycle. In support of this, they found hypomodification of t^6^A37 in mitochondrial tRNA serine isolated from solid tumor xenografts [103]. 

Similarly, agents and exposures that cause macromolecular damage lead to the predictable reprogramming of RNA modifications. The predictive power in tRNA modifications is illustrated with the response of yeast exposed to four oxidants and five alkylating agents [11]. tRNA modification patterns accurately distinguished between the two types of toxicant, with 14 modified ribonucleosides forming the basis for a data-driven model that predicted toxicant chemistry with >80% sensitivity and specificity [11]. tRNA modification subpatterns also distinguished among chemically similar toxicants such as SN1 and SN2 alkylating agents [11]. This distinction further linked to codon-biased translation: SN2-induced increases in m^3^C in tRNA led to selective translation of threonine-rich membrane proteins from genes enriched with ACC and ACT degenerate codons for threonine [11]. tRNA modifications thus serve as predictive biomarkers of exposure.

However, other types of stress response can also promote characteristic epitranscriptomic changes. Looking beyond tRNA, analysis of all cellular RNAs (including mRNA, rRNA, tRNA, and snoRNA) at the nucleoside level has been used to demonstrate that there is broad reprogramming of mRNA-, rRNA- and, tRNA-based modifications in response to osmotic stress. For example, using yeast, it was observed that there were dramatic increases in monomethylated C, representing m^3^C, m^5^C, Cm, and m^4^C in mRNA, rRNA, tRNA, and snoRNA during the osmotic stress response [64]. Also, the tRNA-based wobble U34 modifications mcm^5^U and mcm^5^s^2^U, as well as i^6^A at position 37, were increased in response to osmotic stress. Mechanistically the reason for osmotic stress induced changes in the epitranscriptome could be to promote RNA stabilization, RNA localization as well as translational regulation. Regardless of the mechanistic details, the observation that there are stress-induced changes in modification levels specific to mRNA- and rRNA-based species supports the idea that global epitranscriptomic changes are ingrained in regulatory responses.

The preceding examples with cultured cells and specific stresses illustrate the potential for tRNA modifications to serve as biomarkers of disease and pathology. Defects in RNA modification or their corresponding writers have been linked to cancer, neurodegenerative and neurological defects, and diabetes [4,5,6]. For example, defects in Q levels have been observed in ovarian and lung tumors [104,105]. Defects in the mitochondrial and nuclear writer of dimethylguanosine (m^2^,_2_G), tRNA methyltransferase 1 (TRMT1), have been shown to promote intellectual disabilities, with this epitranscriptomic system linked to redox metabolism [106]. Type II diabetes has also been linked to defects in tRNA modifications, with sequence variants and a mouse model defective in the 2-methylthio-N6-threonylcarbamoyladenosine (ms^2^t^6^A) writer CDKAL1 demonstrating how modification of tRNA for lysine plays an important role in maintaining pancreatic islet function and controlling glucose levels [107,108]. Expression of RNA modification systems have also been linked to cancer survival, as the wobble U modification writer enzyme ALKBH8 has been shown to be required for growth of bladder cancers [109], while the ALKBH3 eraser of m^6^A in RNA is required for survival of non-small cell lung cancer and other cancers [110,111].

## 6. Conclusions and Perspectives

**Figure 4:**
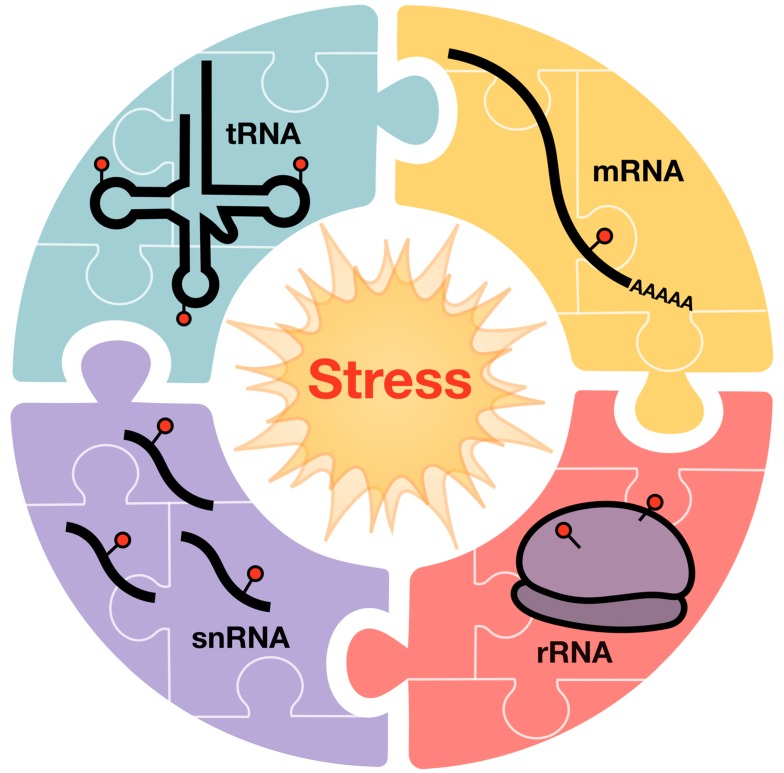
Coordinated changes in epitranscriptomic marks on tRNA, mRNA, rRNA, and snRNA are theorized to drive the translational response to stress, and other physiological responses.

The dynamic regulation of RNA modifications plays an important role in the response of cells to environmental fluctuations and xenobiotic exposures. On average there are 13 modifications in each tRNA and many distinct tRNA isoacceptors in each cell. Global analyses have demonstrated that there are coordinated changes in tRNA modifications, for example, the alkylation-induced increases in mcm^5^U and mcm^5^s^2^U occurring as there are increases in m^3^C and m^7^G [11]. These coordinated changes in epitranscriptomic marks suggest that a program of translational regulation specific to many codon–anticodon pairs is driving the translational response to stress. While almost all tRNA modifications occur in multiple tRNAs at several positions, toxicant-induced changes are affecting very specific positions in individual tRNAs. It is currently not understood, how multiple tRNAs bearing the same modification can be differentially regulated when the sites are targeted by a single enzyme. RNA modification enzymes may rely on the presence of other modifications for the introduction of a modification, as observed for the Dnmt2-mediated introduction of m^5^C, which is stimulated by the presence of Q at position 34 [112]. The investigation whether such crosstalk between modifications is a general phenomenon requires the development of novel techniques allowing the single-base resolution mapping of individual modifications, such as bisulfite sequencing [113]. The presence of certain modifications could lead to structural changes in the three-dimensional structure of tRNA, making other sites more accessible for modification enzymes that were previously hidden. Furthermore, it will not only be essential to unveil how tRNA modifications are coordinately regulated, but also to start integrating the tRNA modification landscape with other epitranscriptomic marks on messenger RNA, such as m^6^A (Figure 4). So far, tRNA, mRNA, and modifications of other RNA types have all been considered as separate entities and their interplay remains completely unstudied. However, some enzymes, like NSUN2, target multiple RNA species for modification, pointing towards the existence of interaction systems between multiple RNA modification systems. It is also important to place translational regulation in the context of a larger program of stress-induced epitranscriptomic changes, which should be regulating tRNA stability, translation initiation, and microRNA-based regulation of transcripts. The observation that there are stress-induced changes in modification levels specific to mRNA- and rRNA-based species supports the idea that global epitranscriptomic changes are ingrained in regulatory responses [64]. Linking tRNA modification-based translational regulation in the context of mRNA modification-based regulation should be an active area of research in the future. There is abundant data to show that the most prevalent mRNA modification—m^6^A—plays dynamic roles in regulating fertility and development, and we suggest that tRNA-based and other epitranscriptomic marks should be important for human development. Further, the identification of m^6^A erasers in the form of demethylase enzymes highlights the dynamic potential of epitranscriptomic marks and suggests that a wide array of tRNA-specific erasers should be present in human cells.

## References

[1] Fulda S., Gorman A.M., Hori O., Samali A. (2010). Cellular Stress Responses: Cell Survival and Cell Death. Int. J. Cell Biol..

[2] Ciccia A., Elledge S.J. (2010). The DNA damage response: Making it safe to play with knives. Mol. Cell.

[3] Schröder M., Kaufman R.J. (2005). The mammalian unfolded protein response. Annu. Rev. Biochem..

[4] Torres A.G., Batlle E., Ribas de Pouplana L. (2014). Role of tRNA modifications in human diseases. Trends Mol. Med..

[5] Zhang X., Cozen A.E., Liu Y., Chen Q., Lowe T.M. (2016). Small RNA Modifications: Integral to Function and Disease. Trends Mol. Med..

[6] Sarin L.P., Leidel S.A. (2015). Modify or die?—RNA modification defects in metazoans. RNA Biol..

[7] Mishima E., Inoue C., Saigusa D., Inoue R., Ito K., Suzuki Y., Jinno D., Tsukui Y., Akamatsu Y., Araki M. (2014). Conformational change in transfer RNA is an early indicator of acute cellular damage. J. Am. Soc. Nephrol..

[8] Kirino Y., Yasukawa T., Ohta S., Akira S., Ishihara K., Watanabe K., Suzuki T. (2004). Codon-specific translational defect caused by a wobble modification deficiency in mutant tRNA from a human mitochondrial disease. Proc. Natl. Acad. Sci. USA.

[9] Chan C.T.Y., Dyavaiah M., DeMott M.S., Taghizadeh K., Dedon P.C., Begley T.J. (2010). A Quantitative Systems Approach Reveals Dynamic Control of tRNA Modifications during Cellular Stress. PLoS Genet..

[10] Patil A., Dyavaiah M., Joseph F., Rooney J.P., Chan C.T.Y., Dedon P.C., Begley T.J. (2012). Increased tRNA modification and gene-specific codon usage regulate cell cycle progression during the DNA damage response. Cell Cycle.

[11] Chan C.T., Deng W., Li F., DeMott M.S., Babu I.R., Begley T.J., Dedon P.C. (2015). Highly Predictive Reprogramming of tRNA Modifications Is Linked to Selective Expression of Codon-Biased Genes. Chem. Res. Toxicol..

[12] Endres L., Begley U., Clark R., Gu C., Dziergowska A., Małkiewicz A., Melendez J.A., Dedon P.C., Begley T.J. (2015). Alkbh8 Regulates Selenocysteine-Protein Expression to Protect against Reactive Oxygen Species Damage. PLoS ONE.

[13] Banerjee R., Chen S., Dare K., Gilreath M., Praetorius-Ibba M., Raina M., Reynolds N.M., Rogers T., Roy H., Yadavalli S.S. (2010). tRNAs: Cellular barcodes for amino acids. FEBS Lett..

[14] Francklyn C.S., Minajigi A. (2010). tRNA as an active chemical scaffold for diverse chemical transformations. FEBS Lett..

[15] Harada F., Sawyer R.C., Dahlberg J.E. (1975). A primer ribonucleic acid for initiation of in vitro Rous sarcarcoma virus deoxyribonucleic acid synthesis. J. Biol. Chem..

[16] Harada F., Peters G.G., Dahlberg J.E. (1979). The primer tRNA for Moloney murine leukemia virus DNA synthesis. Nucleotide sequence and aminoacylation of tRNAPro. J. Biol. Chem..

[17] Mak J., Kleiman L. (1997). Primer tRNAs for reverse transcription. J. Virol..

[18] Isel C., Marquet R., Keith G., Ehresmann C., Ehresmann B. (1993). Modified nucleotides of tRNA(3Lys) modulate primer/template loop-loop interaction in the initiation complex of HIV-1 reverse transcription. J. Biol. Chem..

[19] Zhong J., Xiao C., Gu W., Du G., Sun X., He Q.-Y., Zhang G. (2015). Transfer RNAs Mediate the Rapid Adaptation of *Escherichia coli* to Oxidative Stress. PLoS Genet..

[20] Wek S.A., Zhu S., Wek R.C. (1995). The histidyl-tRNA synthetase-related sequence in the eIF-2 alpha protein kinase GCN2 interacts with tRNA and is required for activation in response to starvation for different amino acids. Mol. Cell. Biol..

[21] Dong J., Qiu H., Garcia-Barrio M., Anderson J., Hinnebusch A.G. (2000). Uncharged tRNA Activates GCN2 by Displacing the Protein Kinase Moiety from a Bipartite tRNA-Binding Domain. Mol. Cell.

[22] Doyle F., Leonardi A., Endres L., Tenenbaum S.A., Dedon P.C., Begley T.J. (2016). Gene- and genome-based analysis of significant codon patterns in yeast, rat and mice genomes with the CUT Codon UTilization tool. Methods.

[23] Mei Y., Yong J., Liu H., Shi Y., Meinkoth J., Dreyfuss G., Yang X. (2010). tRNA binds to cytochrome *c* and inhibits caspase activation. Mol. Cell.

[24] Mei Y., Yong J., Stonestrom A., Yang X. (2010). tRNA and cytochrome *c* in cell death and beyond. Cell Cycle.

[25] Lee Y.S., Shibata Y., Malhotra A., Dutta A. (2009). A novel class of small RNAs: tRNA-derived RNA fragments (tRFs). Genes Dev..

[26] Anderson P., Ivanov P. (2014). tRNA fragments in human health and disease. FEBS Lett..

[27] Maute R.L., Schneider C., Sumazin P., Holmes A., Califano A., Basso K., Dalla-Favera R. (2013). tRNA-derived microRNA modulates proliferation and the DNA damage response and is down-regulated in B cell lymphoma. Proc. Natl. Acad. Sci. USA.

[28] Gebetsberger J., Zywicki M., Künzi A., Polacek N. (2012). tRNA-derived fragments target the ribosome and function as regulatory non-coding RNA in Haloferax volcanii. Archaea.

[29] Sobala A., Hutvagner G. (2013). Small RNAs derived from the 5′ end of tRNA can inhibit protein translation in human cells. RNA Biol..

[30] Davis F.F., Allen F.W. (1957). Ribonucleic acids from yeast which contain a fifth nucleoside. J. Biol. Chem..

[31] Boccaletto P., Machnicka M.A., Purta E., Piątkowski P., Bagiński B., Wirecki T.K., de Crécy-Lagard V., Ross R., Limbach P.A., Kotter A. (2018). MODOMICS: A database of RNA modification pathways. 2017 update. Nucleic Acids Res..

[32] Cantara W.A., Crain P.F., Rozenski J., McCloskey J.A., Harris K.A., Zhang X., Vendeix F.A.P., Fabris D., Agris P.F. (2011). The RNA modification database, RNAMDB: 2011 update. Nucleic Acids Res..

[33] Jackman J.E., Alfonzo J.D. (2013). Transfer RNA modifications: Nature’s combinatorial chemistry playground. Wiley Interdiscip. Rev. RNA.

[34] Agris P.F., Vendeix F.A.P., Graham W.D. (2007). tRNA’s Wobble Decoding of the Genome: 40 Years of Modification. J. Mol. Biol..

[35] Bjork G.R., Wikstrom P.M., Bystrom A.S. (1989). Prevention of translational frameshifting by the modified nucleoside 1-methylguanosine. Science.

[36] Shoffner J.M., Lott M.T., Lezza A.M.S., Seibel P., Ballinger S.W., Wallace D.C. (1990). Myoclonic epilepsy and ragged-red fiber disease (MERRF) is associated with a mitochondrial DNA tRNALys mutation. Cell.

[37] Yasukawa T., Suzuki T., Ishii N., Ohta S., Watanabe K. (2001). Wobble modification defect in tRNA disturbs codon-anticodon interaction in a mitochondrial disease. EMBO J..

[38] Yasukawa T., Suzuki T., Suzuki T., Ueda T., Ohta S., Watanabe K. (2000). Modification Defect at Anticodon Wobble Nucleotide of Mitochondrial tRNAsLeu(UUR) with Pathogenic Mutations of Mitochondrial Myopathy, Encephalopathy, Lactic Acidosis, and Stroke-like Episodes. J. Biol. Chem..

[39] Liu F., Clark W., Luo G., Wang X., Fu Y., Wei J., Wang X., Hao Z., Dai Q., Zheng G. (2016). ALKBH1-Mediated tRNA Demethylation Regulates Translation. Cell.

[40] Fu Y., Jia G., Pang X., Wang R.N., Wang X., Li C.J., Smemo S., Dai Q., Bailey K.A., Nobrega M.A. (2013). FTO-mediated formation of N^6^-hydroxymethyladenosine and N^6^-formyladenosine in mammalian RNA. Nat. Commun..

[41] Jia G., Fu Y., Zhao X., Dai Q., Zheng G., Yang Y., Yi C., Lindahl T., Pan T., Yang Y.-G. (2011). *N*6-methyladenosine in nuclear RNA is a major substrate of the obesity-associated FTO. Nat. Chem. Biol..

[42] Zheng G., Dahl J.A., Niu Y., Fedorcsak P., Huang C.-M., Li C.J., Vågbø C.B., Shi Y., Wang W.-L., Song S.-H. (2013). ALKBH5 is a mammalian RNA demethylase that impacts RNA metabolism and mouse fertility. Mol. Cell.

[43] Zhou J., Wan J., Gao X., Zhang X., Jaffrey S.R., Qian S.-B. (2015). Dynamic m^6^A mRNA methylation directs translational control of heat shock response. Nature.

[44] Xiang Y., Laurent B., Hsu C.-H., Nachtergaele S., Lu Z., Sheng W., Xu C., Chen H., Ouyang J., Wang S. (2017). RNA m^6^A methylation regulates the ultraviolet-induced DNA damage response. Nature.

[45] Li Z., Weng H., Su R., Weng X., Zuo Z., Li C., Huang H., Nachtergaele S., Dong L., Hu C. (2017). FTO Plays an Oncogenic Role in Acute Myeloid Leukemia as a *N*^6^-Methyladenosine RNA Demethylase. Cancer Cell.

[46] Fu L., Guerrero C.R., Zhong N., Amato N.J., Liu Y., Liu S., Cai Q., Ji D., Jin S.G., Niedernhofer L.J. (2014). Tet-mediated formation of 5-hydroxymethylcytosine in RNA. J. Am. Chem. Soc..

[47] Huber S.M., van Delft P., Mendil L., Bachman M., Smollett K., Werner F., Miska E.A., Balasubramanian S. (2015). Formation and abundance of 5-hydroxymethylcytosine in RNA. Chembiochem Eur. J. Chem. Biol..

[48] Huber S.M., van Delft P., Tanpure A., Miska E.A., Balasubramanian S. (2017). 2′-O-Methyl-5-hydroxymethylcytidine: A Second Oxidative Derivative of 5-Methylcytidine in RNA. J. Am. Chem. Soc..

[49] Mauer J., Luo X., Blanjoie A., Jiao X., Grozhik A.V., Patil D.P., Linder B., Pickering B.F., Vasseur J.-J., Chen Q. (2017). Reversible methylation of m^6^A_m_ in the 5′ cap controls mRNA stability. Nature.

[50] Meyer K.D., Jaffrey S.R. (2017). Rethinking m^6^A Readers, Writers, and Erasers. Annu. Rev. Cell Dev. Biol..

[51] Delatte B., Wang F., Ngoc L.V., Collignon E., Bonvin E., Deplus R., Calonne E., Hassabi B., Putmans P., Awe S. (2016). Transcriptome-wide distribution and function of RNA hydroxymethylcytosine. Science.

[52] Patil A., Chan C.T., Dyavaiah M., Rooney J.P., Dedon P.C., Begley T.J. (2012). Translational infidelity-induced protein stress results from a deficiency in Trm9-catalyzed tRNA modifications. RNA Biol..

[53] Nedialkova D.D., Leidel S.A. (2015). Optimization of Codon Translation Rates via tRNA Modifications Maintains Proteome Integrity. Cell.

[54] Tukenmez H., Xu H., Esberg A., Bystrom A.S. (2015). The role of wobble uridine modifications in +1 translational frameshifting in eukaryotes. Nucleic Acids Res..

[55] Paredes J.A., Carreto L., Simões J., Bezerra A.R., Gomes A.C., Santamaria R., Kapushesky M., Moura G.R., Santos M.A.S. (2012). Low level genome mistranslations deregulate the transcriptome and translatome and generate proteotoxic stress in yeast. BMC Biol..

[56] Tuorto F., Lyko F. (2016). Genome recoding by tRNA modifications. Open Biol..

[57] Kirchner S., Ignatova Z. (2014). Emerging roles of tRNA in adaptive translation, signalling dynamics and disease. Nat. Rev. Genet..

[58] Chernyakov I., Whipple J.M., Kotelawala L., Grayhack E.J., Phizicky E.M. (2008). Degradation of several hypomodified mature tRNA species in *Saccharomyces cerevisiae* is mediated by Met22 and the 5′-3′ exonucleases Rat1 and Xrn1. Genes Dev..

[59] Alexandrov A., Chernyakov I., Gu W., Hiley S.L., Hughes T.R., Grayhack E.J., Phizicky E.M. (2006). Rapid tRNA Decay Can Result from Lack of Nonessential Modifications. Mol. Cell.

[60] Cload S.T., Liu D.R., Froland W.A., Schultz P.G. (1996). Development of improved tRNAs for in vitro biosynthesis of proteins containing unnatural amino acids. Chem. Biol..

[61] Esberg A., Huang B., Johansson M.J.O., Byström A.S. (2006). Elevated Levels of Two tRNA Species Bypass the Requirement for Elongator Complex in Transcription and Exocytosis. Mol. Cell.

[62] Droogmans L., Roovers M., Bujnicki J.M., Tricot C., Hartsch T., Stalon V., Grosjean H. (2003). Cloning and characterization of tRNA (m1A58) methyltransferase (TrmI) from Thermus thermophilus HB27, a protein required for cell growth at extreme temperatures. Nucleic Acids Res..

[63] Ishida K., Kunibayashi T., Tomikawa C., Ochi A., Kanai T., Hirata A., Iwashita C., Hori H. (2011). Pseudouridine at position 55 in tRNA controls the contents of other modified nucleotides for low-temperature adaptation in the extreme-thermophilic eubacterium *Thermus thermophilus*. Nucleic Acids Res..

[64] Rose R.E., Pazos M.A., Curcio M.J., Fabris D. (2016). Global Epitranscriptomics Profiling of RNA Post-Transcriptional Modifications as an Effective Tool for Investigating the Epitranscriptomics of Stress Response. Mol. Cell. Proteom..

[65] Basanta-Sanchez M., Temple S., Ansari S.A., D’Amico A., Agris P.F. (2016). Attomole quantification and global profile of RNA modifications: Epitranscriptome of human neural stem cells. Nucleic Acids Res..

[66] Chan C.T.Y., Pang Y.L.J., Deng W., Babu I.R., Dyavaiah M., Begley T.J., Dedon P.C. (2012). Reprogramming of tRNA modifications controls the oxidative stress response by codon-biased translation of proteins. Nat. Commun..

[67] Pestova T.V., Kolupaeva V.G., Lomakin I.B., Pilipenko E.V., Shatsky I.N., Agol V.I., Hellen C.U.T. (2001). Molecular mechanisms of translation initiation in eukaryotes. Proc. Natl. Acad. Sci. USA.

[68] Kozak M. (1983). Comparison of initiation of protein synthesis in procaryotes, eucaryotes, and organelles. Microbiol. Rev..

[69] Basavappa R., Sigler P.B. (1991). The 3 A crystal structure of yeast initiator tRNA: Functional implications in initiator/elongator discrimination. EMBO J..

[70] Anderson J., Phan L., Cuesta R., Carlson B.A., Pak M., Asano K., Björk G.R., Tamame M., Hinnebusch A.G. (1998). The essential Gcd10p-Gcd14p nuclear complex is required for 1-methyladenosine modification and maturation of initiator methionyl-tRNA. Genes Dev..

[71] Kadaba S., Krueger A., Trice T., Krecic A.M., Hinnebusch A.G., Anderson J. (2004). Nuclear surveillance and degradation of hypomodified initiator tRNAMet in *S. cerevisiae*. Genes Dev..

[72] Su D., Chan C.T.Y., Gu C., Lim K.S., Chionh Y.H., McBee M.E., Russell B.S., Babu I.R., Begley T.J., Dedon P.C. (2014). Quantitative analysis of ribonucleoside modifications in tRNA by HPLC-coupled mass spectrometry. Nat. Protoc..

[73] Deng W., Babu I.R., Su D., Yin S., Begley T.J., Dedon P.C. (2015). Trm9-Catalyzed tRNA Modifications Regulate Global Protein Expression by Codon-Biased Translation. PLoS Genet..

[74] Begley U., Dyavaiah M., Patil A., Rooney J.P., DiRenzo D., Young C.M., Conklin D.S., Zitomer R.S., Begley T.J. (2007). Trm9-catalyzed tRNA modifications link translation to the DNA damage response. Mol. Cell.

[75] Chionh Y.H., McBee M., Babu I.R., Hia F., Lin W., Zhao W., Cao J., Dziergowska A., Malkiewicz A., Begley T.J. (2016). tRNA-mediated codon-biased translation in mycobacterial hypoxic persistence. Nat. Commun..

[76] Schaefer M., Pollex T., Hanna K., Tuorto F., Meusburger M., Helm M., Lyko F. (2010). RNA methylation by Dnmt2 protects transfer RNAs against stress-induced cleavage. Genes Dev..

[77] Fu H., Feng J., Liu Q., Sun F., Tie Y., Zhu J., Xing R., Sun Z., Zheng X. (2008). Stress induces tRNA cleavage by angiogenin in mammalian cells. FEBS Lett..

[78] Thompson D.M., Lu C., Green P.J., Parker R. (2008). tRNA cleavage is a conserved response to oxidative stress in eukaryotes. RNA.

[79] Motorin Y., Lyko F., Helm M. (2010). 5-methylcytosine in RNA: Detection, enzymatic formation and biological functions. Nucleic Acids Res..

[80] Suzuki T., Suzuki T. (2014). A complete landscape of post-transcriptional modifications in mammalian mitochondrial tRNAs. Nucleic Acids Res..

[81] Haag S., Warda A.S., Kretschmer J., Günnigmann M.A., Höbartner C., Bohnsack M.T. (2015). NSUN6 is a human RNA methyltransferase that catalyzes formation of m^5^C72 in specific tRNAs. RNA.

[82] Goll M.G., Kirpekar F., Maggert K.A., Yoder J.A., Hsieh C.-L., Zhang X., Golic K.G., Jacobsen S.E., Bestor T.H. (2006). Methylation of tRNAAsp by the DNA Methyltransferase Homolog Dnmt2. Science.

[83] Khoddami V., Cairns B.R. (2013). Identification of direct targets and modified bases of RNA cytosine methyltransferases. Nat. Biotechnol..

[84] Hussain S., Sajini A.A., Blanco S., Dietmann S., Lombard P., Sugimoto Y., Paramor M., Gleeson J.G., Odom D.T., Ule J. (2013). NSun2-Mediated Cytosine-5 Methylation of Vault Noncoding RNA Determines Its Processing into Regulatory Small RNAs. Cell Rep..

[85] Blanco S., Dietmann S., Flores J.V., Hussain S., Kutter C., Humphreys P., Lukk M., Lombard P., Treps L., Popis M. (2014). Aberrant methylation of tRNAs links cellular stress to neuro-developmental disorders. Embo J..

[86] Harada F., Nishimura S. (1972). Possible anticodon sequences of tRNAHis, tRNAAsn, and tRNAAsp from *Escherichia coli*. Universal presence of nucleoside O in the first position of the anticodons of these transfer ribonucleic acid. Biochemistry.

[87] El Yacoubi B., Bailly M., de Crécy-Lagard V. (2012). Biosynthesis and Function of Posttranscriptional Modifications of Transfer RNAs. Annu. Rev. Genet..

[88] Reyniers J.P., Pleasants J.R., Wostmann B.S., Katze J.R., Farkas W.R. (1981). Administration of exogenous queuine is essential for the biosynthesis of the queuosine-containing transfer RNAs in the mouse. J. Biol. Chem..

[89] Katze J.R., Gunduz U., Smith D.L., Cheng C.S., McCloskey J.A. (1984). Evidence that the nucleic acid base queuine is incorporated intact into tRNA by animal cells. Biochemistry.

[90] Pathak C., Jaiswal Y.K., Vinayak M. (2008). Queuine promotes antioxidant defence system by activating cellular antioxidant enzyme activities in cancer. Biosci. Rep..

[91] Wang X., Matuszek Z., Huang Y., Parisien M., Dai Q., Clark W., Schwartz M.H., Pan T. (2018). Queuosine modification protects cognate tRNAs against ribonuclease cleavage. RNA.

[92] Kasai H., Nakanishi K., Macfarlane R.D., Torgerson D.F., Ohashi Z., McCloskey J.A., Gross H.J., Nishimura S. (1976). The structure of Q* nucleoside isolated from rabbit liver transfer ribonucleic acid. J. Am. Chem. Soc..

[93] Lu J., Huang B., Esberg A., Johansson M.J.O., Byström A.S. (2005). The Kluyveromyces lactis gamma-toxin targets tRNA anticodons. RNA.

[94] Ogawa T., Inoue S., Yajima S., Hidaka M., Masaki H. (2006). Sequence-specific recognition of colicin E5, a tRNA-targeting ribonuclease. Nucleic Acids Res..

[95] Jiang Y., Meidler R., Amitsur M., Kaufmann G. (2001). Specific interaction between anticodon nuclease and the tRNALys wobble base11Edited by D. Draper. J. Mol. Biol..

[96] Hopper A.K., Phizicky E.M. (2003). tRNA transfers to the limelight. Genes Dev..

[97] Hopper A.K. (2013). Transfer RNA post-transcriptional processing, turnover, and subcellular dynamics in the yeast Saccharomyces cerevisiae. Genetics.

[98] Foretek D., Wu J., Hopper A.K., Boguta M. (2016). Control of Saccharomyces cerevisiae pre-tRNA processing by environmental conditions. RNA.

[99] Ohira T., Miyauchi K., Sakaguchi Y., Suzuki T., Suzuki T. (2009). Precise analysis of modification status at various stage of tRNA maturation in *Saccharomyces cerevisiae*. Nucleic Acids Symp. Ser..

[100] Nishikura K., De Robertis E.M. (1981). RNA processing in microinjected Xenopus oocytes: Sequential addition of base modifications in a spliced transfer RNA. J. Mol. Biol..

[101] Torres A.G., Piñeyro D., Rodríguez-Escribà M., Camacho N., Reina O., Saint-Léger A., Filonava L., Batlle E., Ribas de Pouplana L. (2015). Inosine modifications in human tRNAs are incorporated at the precursor tRNA level. Nucleic Acids Res..

[102] Gaston K.W., Rubio M.A.T., Spears J.L., Pastar I., Papavasiliou F.N., Alfonzo J.D. (2007). C to U editing at position 32 of the anticodon loop precedes tRNA 5′ leader removal in trypanosomatids. Nucleic Acids Res..

[103] Lin H., Miyauchi K., Harada T., Okita R., Takeshita E., Komaki H., Fujioka K., Yagasaki H., Goto Y.-I., Yanaka K. (2018). CO_2_-sensitive tRNA modification associated with human mitochondrial disease. Nat. Commun..

[104] Baranowski W., Dirheimer G., Jakowicki J.A., Keith G. (1994). Deficiency of Queuine, a Highly Modified Purine Base, in Transfer RNAs from Primary and Metastatic Ovarian Malignant Tumors in Women. Cancer Res..

[105] Huang B.-S., Wu R.-T., Chien K.-Y. (1992). Relationship of the Queuine Content of Transfer Ribonucleic Acids to Histopathological Grading and Survival in Human Lung Cancer. Cancer Res..

[106] Dewe J.M., Fuller B.L., Lentini J.M., Kellner S.M., Fu D. (2017). TRMT1-Catalyzed tRNA Modifications Are Required for Redox Homeostasis to Ensure Proper Cellular Proliferation and Oxidative Stress Survival. Mol. Cell. Biol..

[107] Steinthorsdottir V., Thorleifsson G., Reynisdottir I., Benediktsson R., Jonsdottir T., Walters G.B., Styrkarsdottir U., Gretarsdottir S., Emilsson V., Ghosh S. (2007). A variant in CDKAL1 influences insulin response and risk of type 2 diabetes. Nat. Genet..

[108] Wei F.-Y., Suzuki T., Watanabe S., Kimura S., Kaitsuka T., Fujimura A., Matsui H., Atta M., Michiue H., Fontecave M. (2011). Deficit of tRNA(Lys) modification by Cdkal1 causes the development of type 2 diabetes in mice. J. Clin. Investig..

[109] Shimada K., Nakamura M., Anai S., De Velasco M., Tanaka M., Tsujikawa K., Ouji Y., Konishi N. (2009). A Novel Human AlkB Homologue, ALKBH8, Contributes to Human Bladder Cancer Progression. Cancer Res..

[110] Ueda Y., Ooshio I., Fusamae Y., Kitae K., Kawaguchi M., Jingushi K., Hase H., Harada K., Hirata K., Tsujikawa K. (2017). AlkB homolog 3-mediated tRNA demethylation promotes protein synthesis in cancer cells. Sci. Rep..

[111] Tasaki M., Shimada K., Kimura H., Tsujikawa K., Konishi N. (2011). ALKBH3, a human AlkB homologue, contributes to cell survival in human non-small-cell lung cancer. Br. J. Cancer.

[112] Ehrenhofer-Murray A.E. (2017). Cross-Talk between Dnmt2-Dependent tRNA Methylation and Queuosine Modification. Biomolecules.

[113] Schaefer M., Pollex T., Hanna K., Lyko F. (2009). RNA cytosine methylation analysis by bisulfite sequencing. Nucleic Acids Res..

